# X-Linked EGFP Reporter as a Tool to Examine X-Chromosome Inactivation in Mouse Embryos and Embryonic Fibroblasts

**DOI:** 10.3390/biom16030375

**Published:** 2026-03-02

**Authors:** Martin Urbán, András Ecker, Roland Imre Tóth, Bence Lázár, Szilárd Bodó, Elen Gócza

**Affiliations:** 1 Institute of Genetics and Biotechnology, Hungarian University of Agriculture and Life Sciences, Szent-Györgyi Albert Str. 4, 2100 Gödöllő, Hungary; urban.martin@ttk.hu (M.U.); ecker.andras@uni-mate.hu (A.E.); gyongybagoly89@gmail.com (R.I.T.); lazar.bence@uni-mate.hu (B.L.); 2 Agribiotechnology and Precision Breeding for Food Security National Laboratory, 2100 Gödöllő, Hungary; 3 Institute of Molecular Life Sciences, Center of Excellence of The Hungarian Academy of Sciences, HUN-REN Research Centre for Natural Sciences, 1117 Budapest, Hungary; 4 Institute of Aquaculture and Environmental Safety, Hungarian University of Agriculture and Life Sciences, Szent-Györgyi Albert Str. 4, 2100 Gödöllő, Hungary; 5 National Centre for Biodiversity and Gene Conservation, 2100 Gödöllő, Hungary; 6 Department of Animal Husbandry Sciences, Hungarian University of Agriculture and Life Sciences, 2100 Gödöllő, Hungary; bodo.szilard@uni-mate.hu

**Keywords:** X chromosome inactivation, mouse embryology, cell culture, fibroblast

## Abstract

This study aimed to establish a model for investigating X chromosome inactivation using transgenic mouse strains expressing green fluorescent protein (GFP). The D4/XGFP-Tg (XGFP) strain carries the GFP transgene on the X chromosome; therefore, due to random X chromosome inactivation, female offspring from crosses between XGFP males and CD-1 females exhibit mosaic GFP expression. In contrast, the B5/EGFP-Tg (EGFP) strain harbours autosomal integration of the same reporter construct, resulting in uniform GFP expression in progenies. Analysis of CD-1 × XGFP attached blastocysts revealed strong GFP expression in giant trophoblast cells and primordial germ cells (PGCs) at E6.5, demonstrating paternal X-chromosome reactivation. In 14.5-day-old CD-1 × XGFP female embryos and CD-1 × EGFP embryos, intense CAG promoter-driven GFP signals were detected in the brain, heart, gonads, somites, and limbs. In line with random X-chromosome inactivation, only 56% of embryonic fibroblast cells, derived from CD-1 × XGFP female embryos, exhibited GFP expression. These findings validate that CD-1 × XGFP mice represent a valuable in vivo model for studying X chromosome inactivation during early embryonic development and PGC specification. Furthermore, CD-1 × XGFP embryonic fibroblasts represent a valuable in vitro model for investigating the molecular mechanisms governing X-chromosome activation and inactivation.

## 1. Introduction

X chromosome inactivation is a process of dosage compensation. It leads to the partial or complete silencing of one X chromosome during cell division [1]. In mouse embryos, both X chromosomes are transcriptionally active during zygotic genome activation (ZGA) at the 2-cell stage. Imprinted inactivation of the paternal X chromosome (Xp) begins at the 4-cell stage and is maintained in extraembryonic lineages. In the inner cell mass (ICM), the Xp is reactivated in cells that give rise to the embryo proper. After implantation, the epiblast undergoes random X chromosome inactivation (XCI), independent of parental origin, thereby establishing the future inactive X chromosome (Xi) [2,3,4].

Several mouse models have been developed to observe X chromosome inactivation. Tan used a mouse model with an *E. coli* lacZ gene integrated into the X chromosome [5]. However, this model cannot detect X inactivation in live tissue or cell cultures. To observe random inactivation, two transgenic mouse strains expressing green fluorescent protein (GFP) were applied: D4/XGFP-TG and B5/EGFP-Tg [6,7]. These strains were created by Anna-Katerina Hadjantonakis in 1998 as D4-XGFP and B5-EGFP, respectively. Both used the same transgene construct [7] but differed in the insertion site. In the D4-XGFP strain, the transgene was integrated on the X chromosome. In the B5-EGFP strain, it was integrated into the autosome [7]. Both strains were crossed to the CD-1 background to form the XGFP and EGFP mouse strains used in our study. Initially, the D4-XGFP mouse strain was used for non-invasive in vitro diagnosis. When blastocyst-stage (E3.5) embryos from a cross between XGFP transgenic males and wild-type CD-1 females were collected, separated into green fluorescent (prospective female) and non-green fluorescent (prospective male) pools, and transferred to recipient females. These females gave birth to single-sex, single-colour litters, in which only pups from the female-only litters exhibited green fluorescence [6]. CD-1 × EGFP embryos showed intense GFP expression in the inner cell mass.

In our study using CD-1 × XGFP mouse embryos, we monitored the paternal X-chromosome inactivation in preimplantation mouse embryos, attached blastocysts, 14.5-day-old embryos, and embryonic fibroblasts. We highlighted that CD-1 × XGFP embryonic fibroblast cells could be a good model for investigating X-chromosome inactivation and reactivation.

## 2. Materials and Methods

### 2.1. Animals

In our experiment, female CD-1 mice (*Mus musculus*) were used. CD-1 mice were obtained from Charles-Rivers Laboratory (Wilmington, MA, USA, 022). CD-1 is an albino mouse strain used as a general multipurpose model. We paired the CD-1 females with males from D4/XGFP-Tg or B5/EGFP-Tg (The Jackson Laboratory, Bar Harbour, ME, USA, 003116) mouse strains. These two mouse strains contain the same EGFP (enhanced green fluorescent protein) transgene construct. The difference is in the integration site of the transgenes. The original mouse lines were backcrossed to the CD-1 background. Our institute then established the XGFP and EGFP mouse lines. Our experiments demonstrated that these mouse lines, even after more than 10 years since their establishment, still exhibit the same characteristics as the original strains.

Animals were housed in groups with ad libitum access to food and water under standard conditions, with a 12 h light/12 h dark cycle (06:00–18:00) at 22 °C. Throughout the experiments, all efforts were made to minimise animal pain. Euthanasia was performed by cervical dislocation.

The day of vaginal plug detection was designated as embryonic day E0.5 (0.5 dpc). For in vitro manipulation and immunostaining, E3.5 and E4.5 embryos were flushed from the uterus of pregnant female mice after euthanasia.

Embryos at 14.5 days of gestation were recovered from the uterus of pregnant females after euthanasia and were used for the establishment of mouse embryonic fibroblast (MEF) cultures. As the mice were naturally paired and no hormonal treatment was applied, no special permission was required for embryo recovery.

### 2.2. Embryo Collection

The animals used were CD-1, XGFP, and EGFP mice, and all embryos were handled in agreement with the Hungarian Code of Practice for the Care and Use of Animals for Scientific Purposes. Embryo collections were performed as previously published [8].

The experiments involving mice in this study were conducted in accordance with the following authorizations and ethical approvals: Establishment of mouse embryonic fibroblast lines (22.1/1131/003/2008/2008.06.16); maintenance of mouse lines (PEI/001/303-4/2013/2013.04.25); Authorisation of genetic engineering activity in safety class I (106685/4/2005/2005/2005.06.23).

### 2.3. Preimplantation Mouse Embryo Cultivation

Following the euthanasia of the female animals, the uterus was removed using sterile surgical instruments. The uterus was then placed in a 1.5 mL (Greiner Bio-one, Billings, MT, USA, E100700C) centrifuge tube filled with 1 mL M2 (Sigma, St. Louis, MO, USA, M7167) medium, which was held on a 37 °C warming plate. Following dissection, the organs were freed from the surrounding adipose tissue and mesometrium and transferred into a fresh drop of M2 medium. For the washing procedure, a syringe with a 26-gauge needle and a polished tip, filled with 1 mL of M2 medium, was used. Following the washing step, 3.5 dpc embryos were collected using a glass capillary and cultured in KSOM medium for 1 day at 37 °C with 5% CO_2_.

After one day, the embryos were transferred to Vitrolife cell slides (Vitrolife, Göteborg, Sweden, I-P8-2106-1) treated with 0.1% gelatine (Sigma, St. Louis, MO, USA, G1890). KO-DMEM cell culture medium was used for further culture. The medium contained 76% Knock-out DMEM (Gibco, Billings, MT, USA, 10829-012), 1% Glutamax supplement 100× (Gibco, Billings, MT, USA, 35050-038), 1% Penicillin-Streptomycin mixture (Gibco, Billings, MT, USA, 15070-063), 1% nonessential amino acids 100× (Gibco, Billings, MT, USA, 11140-050), 0.1% B-mercaptoethanol (Gibco, Billings, MT, USA, 31350010), 20% ES Cell quality foetal bovine serum (FBS) (Gibco, Billings, MT, USA, 10439-024) and 0.1% mouse LIF (Sigma, St. Louis, MO, USA, ESG1106). The embryos were cultured for three days until the formation of ICM clumps, after which they were fixed in 4% paraformaldehyde (PFA; Fluka, Buchs, Switzerland; 30525-89-4) prior to immunostaining.

### 2.4. Mouse Embryonic Fibroblast Isolation and Cultivation

Embryos at 14.5 days of gestation were used to establish embryonic fibroblast cultures. Following the collection of the embryos from the uterus, they were placed in a 37 °C DPBS solution (GIBCO, Billings, MT, USA, 14190-250), which was used to remove blood from the embryos. Subsequently, the placenta, yolk sac, and amniotic sac were removed using sterile forceps and scissors. The embryos were transferred into fresh DPBS, after which the head and internal organs were surgically removed from the embryos. Subsequently, the embryos were individually transferred into a 50 mL Falcon tubes containing 2 mL of 0.25% Trypsin-EDTA (GIBCO, Billings, MT, USA, 25200-056) solution. Using sterile scissors, the embryos were cut into small pieces of approximately 1 mm in size and digested for 30 min in a Thermomixer (Eppendorf, Hamburg, Germany) at 37 °C and 600 rpm (revolutions per minute). In a sterile laminar flow hood, the suspension containing the embryonic tissue pieces was gently resuspended to enhance digestion efficiency. After a 30 min incubation, enzymatic activity was terminated by the addition of 4 mL of FM, and the cell suspension was subsequently filtered through a 70 µm cell strainer (Falcon, Mexico City, Mexico, 104386). Centrifugation was then performed at 326 G, 4 °C for 5 min was then performed, after which the supernatant was removed using a Pasteur pipette. Meanwhile, the T25 culture flasks (Corning, Corning, NY, USA, 430639) were prepared. The remaining cells were then resuspended in 1 mL of FM and transferred to the culture flasks. The medium contained 86% KnockOut-DMEM/F-12(1X) (GIBCO, Billings, MT, USA, 12660-012), 1% Embryomax Nucleosides (Merck, Darmstadt, Germany, ES-008-D), 1% Glutamax supplement 100×, 1% Penicillin-Streptomycin, 1% Nonessential amino acids, 10% FBS. The cells were then further cultured in an incubator at 37 °C in a humidified, 5% CO_2_ environment. After 24 h, the FM was removed using a Pasteur pipette and replaced with fresh medium. The fibroblast culture medium was replaced with a fresh medium every two days.

Fibroblasts were cultured until they reached 80% confluency. The cultures were passaged twice before cryopreservation. During passaging of the embryonic fibroblast cultures, the culture medium was aspirated using a Pasteur pipette connected to a vacuum pump. The cells were subsequently washed with prewarmed (37 °C) DPBS. After removal of the DPBS, 2 mL of trypsin–EDTA solution was added, and the cells were incubated for 7 min to allow enzymatic detachment. The digestion was stopped by the addition of FM in a 1:1 ratio to the cell suspension. The suspension was collected using a serological pipette and transferred into a 50 mL centrifuge tube (Corning, Glendale, AZ, USA). The cells were centrifuged at 326 G for 7 min at 4 °C. Subsequently, the supernatant was removed with a Pasteur pipette connected to a vacuum pump. The cell pellets were resuspended in 2 mL of fibroblast culture medium and seeded into either two T25 flasks (Corning, 430639) containing 5 mL or T75 flasks (Corning, 430641U) containing 9 mL of FM, depending on cell density. Cultures were maintained at 37 °C in a humidified atmosphere of 5% CO_2_ and 95% relative humidity.

During the experiment, a total of 57 CD-1 × XGFP, 14 CD-1 × EGFP, 8 XGFP × XGFP, and 24 EGFP × EGFP embryos were used. We established a total of 8 CD-1 × XGFP, 4 CD-1 × EGFP, 8 XGFP × XGFP, and 4 EGFP × EGFP mouse embryonic fibroblast cell lines. These cell lines were expanded until four T75 flasks reached complete confluence. The cultures were frozen in cryotubes, each containing 1.5 × 10^5^ cells. GFP expression in the cultures was examined using Nanoentek Arthur fluorescent cell counter (NanoEntek, Soeul, Republic of Korea) and under Leica M205 FCA fluorescence stereo equipped with a DFC7000-T camera and Leica TCS SP8 confocal microscopes (Leica, Wetzlar, Germany). During the study, we established a total of eight CD-1 × XGFP, four CD-1 × EGFP, eight XGFP × XGFP, and four EGFP × EGFP mouse embryonic fibroblast cell lines. Each line was expanded until four T75 flasks reached full confluence, after which all newly established MEF cultures were cryopreserved. The frozen vials are stored at −150 °C in a Panasonic ultra-low temperature freezer in our institutional gene bank for long-term storage. The cell lines were not deposited in a public biobank, as they were generated exclusively for use in the present study to investigate X-chromosome inactivation.

### 2.5. Freezing of the Embryonic Fibroblast Cultures

Before freezing the embryonic fibroblast cultures, the freezing medium was freshly prepared. The 2× freezing medium consists of FM (60%), FBS (20%) and DMSO (20%) (Sigma, St. Louis, MO, USA, D2650). Subsequently, the final 2× freezing medium was filtered through a 0.2 µm syringe filter (Merck Millipore, Merck, Darmstadt, Germany, SLGPM33RS) and stored at 4 °C until required. After centrifugation, the supernatant was removed, and the cells were resuspended in 2.5 mL of FM medium. Then 2.5 mL of 2× freezing medium was added to the centrifuge tube. After gently mixing the cell suspension containing FM medium with the added 2× freezing medium, 1 mL of cell suspension was added to each freezing tube. The freezing tubes were then placed in a BioCool freezer box at −70 °C for 24 h. After 24 h, the freezing tubes were transferred to a temperature of −150 °C in a Panasonic Ultralow Freezer.

### 2.6. Immunostaining of the Attached Blastocysts

Blastocysts at E4.5 were placed onto a mitomycin-treated CD-1 mouse embryonic fibroblast feeder cell layer at 80% confluency. Before mitomycin treatment, the feeder cells were plated onto a 0.1% gelatin-coated surface. The blastocysts attached to the feeder cell layer on the first day. The attached blastocysts were fixed on the second day of the culture in 4% PFA for ten minutes. After fixation, the PFA was collected in a separate container, and the cells were washed three times for five minutes in 250 µL of 0.01% BSA (Sigma, St. Louis, MO, USA, A3311)—PBS. Subsequently, to prevent the non-specific binding, a blocking solution was added (100 µL per well). The blocking solution consisted of 0.1% BSA, 2.5% donkey serum, and 0.1% Triton X-100 in PBS. Following a 45 min incubation period, the blocking solution was removed, and 100 µL of 0.1% BSA-PBS containing the primary antibody against P63 (BioMedica, Budapest, Hungary, A31572) at the appropriate concentration (1:100 dilution in 0.1% BSA-PBS) was added to each well. After overnight incubation at 4 °C, the primary antibody solution was removed, and the cells were washed three times with 100 µL of 0.01% BSA-PBS per well. The secondary antibody, anti-rabbit IgG Alexa Fluor™ 555 (Life Technologies/Molecular Probes, Carlsbad, CA, USA, T3605), was diluted 1:500 in 0.1% BSA-PBS. Then, 100 µL of the staining solution was added to each well and incubated in the dark at 37 °C for 60 min. The staining solution was subsequently removed, and the wells were washed three times with 0.01% BSA-PBS for 10 min each. From the solution, 100 µL per well was discarded and allowed to act for 15 min. Subsequently, the Topro-3 solution was removed from the wells, and the cells were washed three times with 0.01% BSA-PBS, adding 250 µL per well at each step for 10 min. Following the final washing step, 10 µL of Vectashield mounting medium (Vector, Burlingame, CA, USA, H-1000) was used to cover the samples. The samples were investigated with a Leica TCS SP8 Confocal Microscope.

### 2.7. Sex Determination of the Mouse Embryonic Fibroblast Cultures

The DNA was extracted from the cells collected from the mouse embryonic fibroblast cultures using the phenol-chloroform DNA isolation protocol. The isolated DNA samples were subjected to analysis using Zfx-L, Zfx-R, Zfy-L, and Zfy-R primers, which were diluted to a concentration of 10 µM for the polymerase chain reaction (PCR). The polymerase chain reaction was conducted using MyTaq Red Mix (Bioline Reagents Ltd., London, UK, BIO-25044). The PCR products were analysed by electrophoresis on a 1.5% agarose gel (Lonza, Basel, Switzerland) treated with ethidium bromide (Sigma, St. Louis, MO, USA, E1510-10ML). The reaction was conducted at 90 volts for 30 min. The DNA bands were then examined under ultraviolet light and photographed. The expected sizes of the PCR products were 104 bp (Zfx) and 299 bp (Zfy), respectively (Carstea et al., 2007 [9]) (Table 1).

### 2.8. AI Usage Disclosure

During the preparation of this manuscript, the authors used ChatGPT (OpenAI), powered by GPT-5.2, for language-editing support, and Grammarly Premium for Mac for grammar correction. The authors reviewed and edited the generated output and take full responsibility for the content of this publication.

## 3. Results

### 3.1. Examination of the EGFP Expression Pattern in Preimplantation Embryos

**Figure 1:**
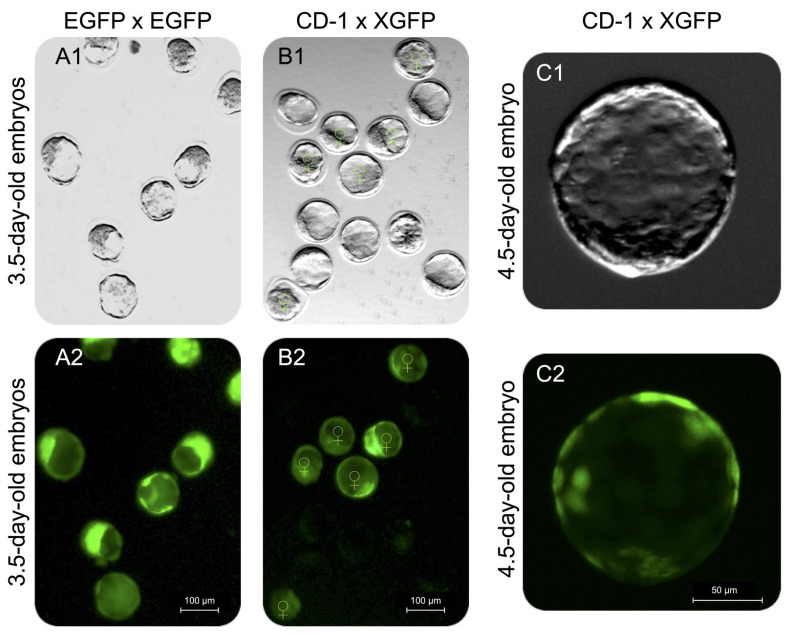
During examination, E3.5 EGFP × EGFP embryos (**A1**) showed intense GFP expression in the inner cell mass (ICM) (**A2**). In contrast, E3.5 CD-1 × XGFP embryos, GFP expression was observed only in half of the embryos (**B1**,**B2**). At E4.5, CD-1 × XGFP embryos (**C1**) exhibited faint and mosaic GFP expression in the ICM (**C2**).

**Figure 2:**
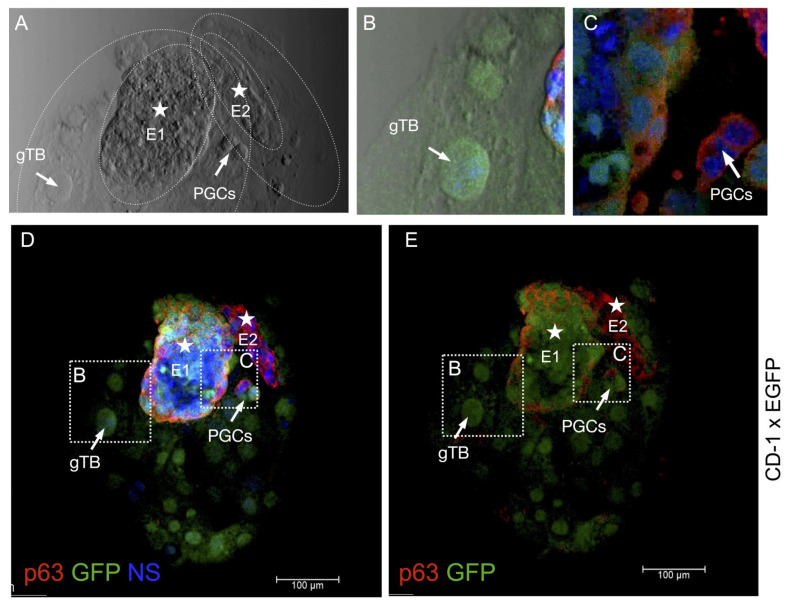
E6.5 embryos (**E1**,**E2**) were attached to the mouse embryonic fibroblast feeder layer (MEF) (**A**,**D**,**E**). Large, round GFP-positive cells (green color) were observed outside the inner cell mass (ICM) (**B**,**C**). After immunostaining, these cells were identified as giant trophoblast cells (gTB) based on their GFP expression (only green expression) (**B**) and as primordial germ cells (PGCs) (**C**) based on their P63 expression (green and red color). Nuclear staining (NS) is blue. The ICM clumps of the attached CD1 × EGFP embryos (E1 and E2) are indicated with one asterisk.

Embryos were flushed from the uteri at E3.5 days post coitum (dpc) (Table 1). These embryos were analysed at E3.5 (Figure 1(A1,A2,B1,B2)), E4.5 (Figure 1(C1,C2)) and E6.5 (Figure 2A–E, Movies S1 and S2).

In the EGFP mouse line, a total of 11 high-quality blastocysts were recovered from five females (Table 1). All recovered EGFP embryos exhibited strong and uniform fluorescence (Figure 1(A1,A2)).

In contrast, embryos obtained from CD-1 females crossed with XGFP males only six (Figure 1(B2)) of the thirteen (Figure 1(B1)) E3.5 blastocysts showed detectable GFP expression.

At E4.5 days post coitum, CD-1 × XGFP embryos (Figure 1(C1)) exhibited faint and mosaic GFP expression in the inner cell mass (ICM) (Figure 1(C2)).

**Figure 3:**
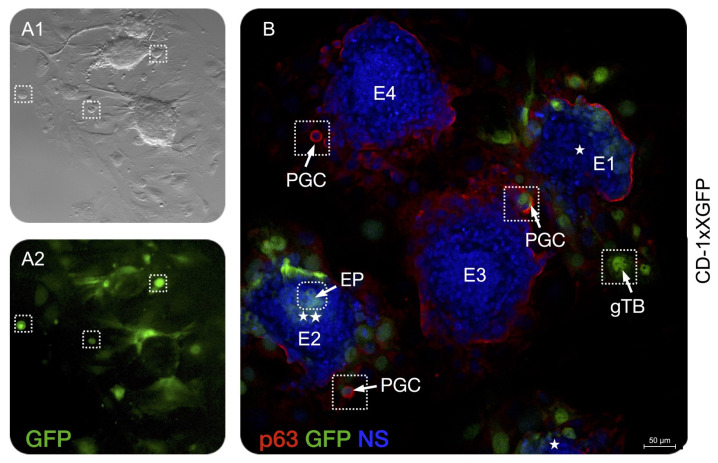
E6.5 mouse embryos (**E1**,**E2**,**E3**,**E4**) were attached to the MEF feeder layer (**A1**,**A2**,**B**). During examination, high GFP expression was observed in the trophoblast (**A2**). In addition, some large, round cells exhibited higher levels of GFP expression ((**B**), gTB—green expression). After immunostaining, these cells did not show P63 positivity ((**B**), gTB). In PGCs it was visible P63 expression (red color) (**B**). Nuclear staining (NS) is blue. The ICM clump of the attached E1 embryo is indicated with one asterisk, while the attached ICM clump of the E4 embryo is marked with two asterisks.

**Figure 4:**
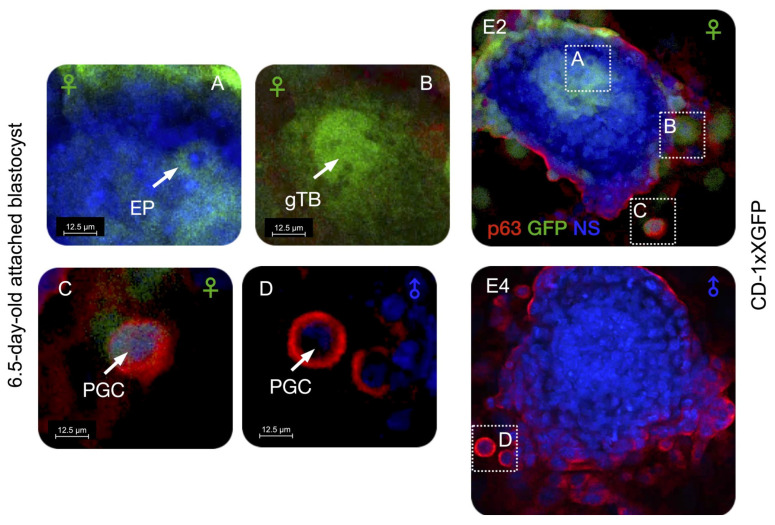
Shows higher magnification images of the (**E2**,**E4**) embryos presented in Figure 3. E6.5, female mouse embryo (**E2**) and male mouse embryo (**E4**) attached to the MEF feeder layer. GFP expression was observed in the female embryo (**E2**), both in the trophoblast (**B**) and epiblast (**A**). In addition, some large, round cells exhibited high levels of GFP expression (gTB—green color). After immunostaining, these cells did not show P63 positivity (**B**). PGCs show P63 expression (red color) (**C**,**D**). Nuclear staining (NS) is blue.

At E6.5, mosaic GFP expression was observed within the ICM, together with marked differences in fluorescence intensity among embryos (Figure 2 and Figure 3A,B). This variability can be attributed to two main factors. First, the X-linked GFP transgene is expressed at lower intensity than the autosomally integrated GFP reporter (Figure 2, Figure 3 and Figure 4). Second, random X-chromosome inactivation in female embryos results in mosaic GFP expression patterns (Figure 3 and Figure 4).

### 3.2. Investigation of the Attached Blastocyst

Following microscopic analysis, embryos were transferred into culture dishes coated with 0.1% gelatine and further cultured in embryonic stem cell culture medium (KO-EM). Upon attachment, changes in GFP expression were detected in the developing extraembryonic tissues linages of both mouse lines (Figure 2, Figure 3 and Figure 4). In EGFP embryos, fluorescence intensity was reduced but remained clearly detectable (Figure 2(E1,E2)); Supplementary Movie S1), whereas in XGFP embryos (Figure 3(E1–E4)) only minimal GFP expression was observed in extraembryonic tissues (Figure 3 and Figure 4, Supplementary Movie S2). In contrast, GFP-positive cells persisted in the ICM in both lines (Figure 2 and Figure 3).

To further characterize the spatial distribution of GFP-expressing cells, attached embryos were subsequently subjected to immunostaining.

Immunostaining of attached embryos was performed using a stem cell–specific P63 antibody, and nuclei were counterstained with TO-PRO-3. In attached female CD-1 × XGFP embryos, giant trophoblast cells exhibiting strong GFP expression were observed, consistent with previous reports by Hadjantonakis et al. [10]. (Figure 4B). These large, flat cells lacked P63 immunoreactivity, similar to the other trophectodermal cells. Apart from the giant trophoblasts (Figure 2B and Figure 3B), the remaining trophoblast cells did not display detectable GFP expression, in agreement with the literature.

In addition, a small population of round cells located at the periphery of the attached ICM showed strong GFP and P63 co-expression (Figure 2C, Figure 3B and Figure 4C,D). Besides giant trophoblasts and PGCs, GFP fluorescence was also detected in epiblast cells of female CD-1 × XGFP embryos (Figure 4A).

In contrast, GFP expression was not detected in attached CD-1 × XGFP male embryos (Figure 3(E3,E4) and Figure 4(E4)) as expected due to the absence of the X-linked transgene. Nevertheless, based on cell morphology and P63 positivity, round cells migrating from the inner cell mass (ICM) could still be identified and are presumed to represent PGCs (Figure 4C,D).

### 3.3. Investigation of 14.5-Day-Old Embryos

**Figure 5:**
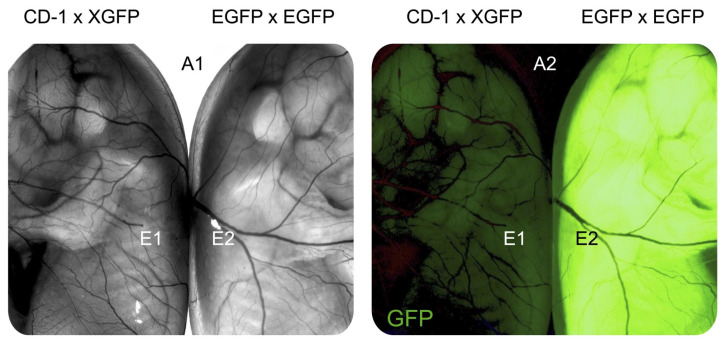
The embryos were examined using a Leica stereomicroscope under identical imaging settings. CD-1 × XGFP embryo (**E1**) exhibited lower levels of GFP expression compared with CD-1 × EGFP embryo (**E2**). (**A1**) (bright-field), (**A2**) (fluorescence microscope).

Embryos were recovered from females at E14.5 (Table 2). Comparative analysis of CD-1 × XGFP and CD-1 × EGFP embryos revealed marked differences in GFP fluorescence intensity. Under identical imaging conditions, CD-1 × XGFP embryos displayed weak and mosaic GFP expression, whereas CD-1 × EGFP embryos exhibited strong and uniform fluorescence, reflecting the X-linked versus autosomal integration of the reporter transgene (Figure 5(A1,A2)).

**Figure 6:**
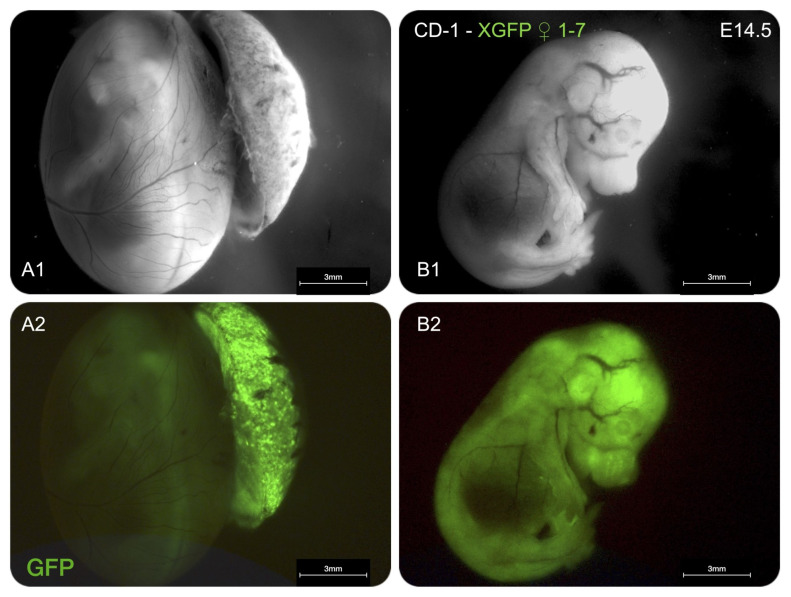
E14.5 CD-1 × XGFP-1-7 female mouse embryo within the yolk sac is shown in (**A1**,**A2**). Intense GFP expression was observed in the placental tissue, specifically in the fetal layer (**A2**). After removal of the extraembryonic tissues (**B1**,**B2**), mosaic GFP expression was detected in the embryonic tissues. (**A1**,**B1**) (bright-field), (**A2**,**B2**) (fluorescence microscope).

**Figure 7:**
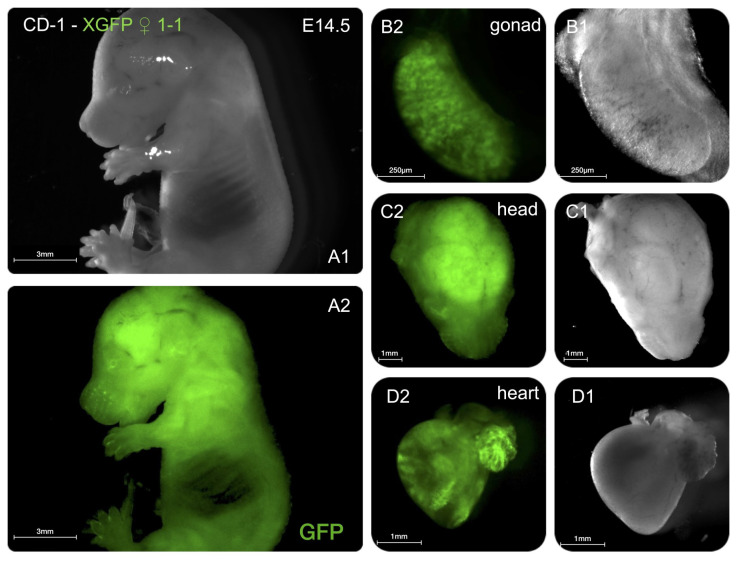
After the removal of the extra embryonic tissues, we observed the GFP expression in E14.5 CD1 × XGFP-1-1 female embryo (**A2**). There was a more intense GFP expression in the gonads (**B1**,**B2**), in the brain (**C1**,**C2**) and in the heart (**D1**,**D2**). (**A1**–**D1**) (bright-field), (**A2**–**D2**) (fluorescence microscope).

In 14.5-day-old CD-1 × XGFP embryos (Figure 6(B1,B2) and Figure 7(A1,A2)), strong but mosaic GFP expression was observed in the placenta (Figure 7(A1,A2)). These GFP-positive cells belong to the foetal layer of the placenta. In addition, strong GFP signals were detected in the brain, heart, and germ cells within the gonads (Figure 7B–D).

Despite the overall strong fluorescence, mosaic GFP patterns were clearly detectable in CD-1 × XGFP embryos, particularly in the myocardium (Figure 7(D1,D2)), consistent with random X-chromosome inactivation. Strong GFP expression was also observed in the limb buds (Figure 6(A2)), especially in the interdigital mesenchyme, where extensive cell proliferation and tissue remodelling occur during digit separation. Furthermore, intense GFP signals were detected in the nasal region at sites corresponding to developing hair follicle placodes, which are characterized by high mitotic activity (Supplementary Figure S1).

During the dissection of 14.5-day-old embryos, clear differences in GFP intensity were also evident between genotypes. When CD-1 × XGFP embryos were compared with EGFP × EGFP embryos, significantly stronger and more homogeneous GFP expression was observed in the latter under identical microscope settings (Figure 5 and Figure S1). Similarly, CD-1 × EGFP embryos exhibited markedly higher and more uniform GFP fluorescence than CD-1 × XGFP embryos, further confirming the effect of autosomal versus X-linked transgene localization on expression level and mosaicism.

Overall, these results demonstrate that while the CAG promoter drives strong GFP expression in multiple embryonic tissues, X-linked integration results in mosaic expression patterns due to random XCI, whereas autosomal integration leads to uniformly high reporter activity throughout development. These more intense expression rates were caused by the CAG promoter in the transgene construct.

### 3.4. Derivation of Mouse Embryonic Fibroblast Cultures

MEF cultures were established from embryos at E14.5. Prior to cell isolation, GFP expression in the embryos was examined using a Leica fluorescence stereomicroscope (Table 3). MEF lines were successfully established and expanded by serial passaging. For immunostaining and GFP expression analysis, a subset of cells was seeded onto 0.1% gelatine-coated slides (Figure S2).

**Figure 8:**
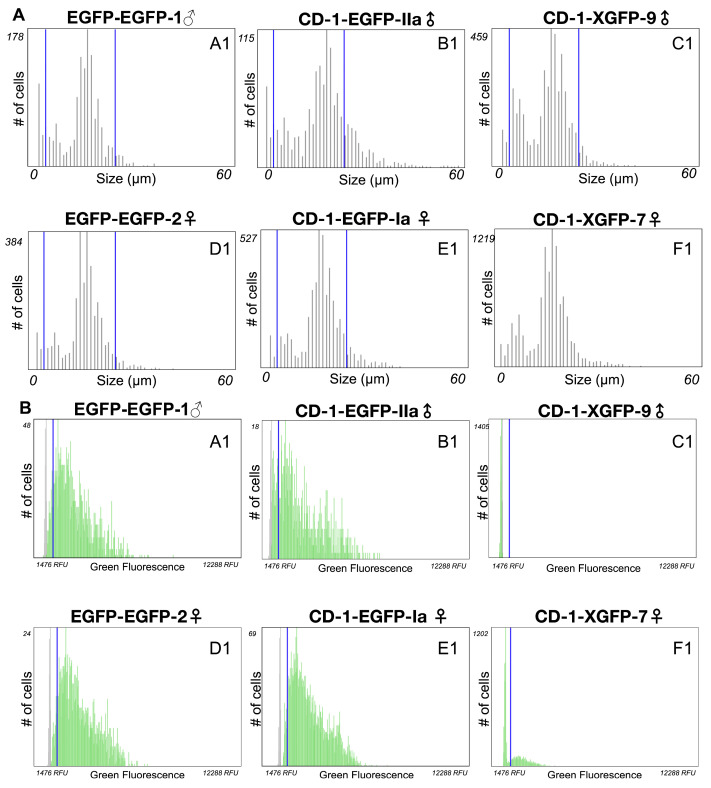
In the graphs, the cell size distribution of fibroblasts measured with the NanoEntek Arthur Fluorescent Cell Counter is shown (**A**). On the bottom part (**B**) GFP expression levels in the different cell lines are displayed. (**A**) The figure shows the cell number according to cell size (µm). (**B**) The figure shows the cell number according to cell fluorescence intensity (RFU). (**A1**,**B1**,**C1**,**D1**,**E1**,**F1**) represent the analysed MEF lines.

Quantitative assessment of GFP-positive cells was performed using an Arthur fluorescence cell counter (Table 4 and Figure 8). In male-derived cell lines, GFP expression was detected in 91% of cells in the EGFP–EGFP-1♂ line (Figure 8) and in 86% of cells in the CD-1 × EGFP-IIa♂ line (Table 4 and Figure 8), whereas no GFP-positive cells were observed in the CD-1 × XGFP-9♂ line (Table 4 and Figure 8). In female-derived cultures, 94% of cells in the EGFP–EGFP-2♀ line (Table 4 and Figure 8) and 95% in the CD-1 × EGFP-Ia♀ line (Table 4 and Figure 8) expressed GFP. In contrast, the CD-1 × XGFP-7♀ line (Table 4 and Figure 8) showed a markedly lower proportion of GFP-positive cells, with only 56% of cells exhibiting fluorescence (Figure S2).

## 4. Discussion

During early mouse embryogenesis, the first XCI event occurs at the 2–4-cell stage, when the paternal X chromosome becomes transcriptionally silenced [11]. This imprinted inactivation is maintained in the trophectoderm lineage; however, a reactivation of the paternal X chromosome has been reported in giant trophoblast cells [10]. Consistent with this, we observed a pattern suggesting paternal X reactivation in these cells.

In the attached ICM, random XCI was evident, indicating that the ICM-derived cells had initiated the transition to the epiblast-like state, characterized by loss of imprinted XCI. Furthermore, immunostaining for P63 revealed the presence of PGCs migrating from the ICM clump. P63 and its isoforms are known to be expressed during gonadal development and serve as reliable markers for identifying PGCs [12]. These P63-positive cells exhibited markedly stronger GFP expression compared to the surrounding ICM, supporting their distinct developmental identity. Based on their morphology, marker expression, and position, these cells were identified as PGCs, which migrate from the epiblast at this developmental stage [13].

At later stages of placental development, trophoblast progenitors undergo additional cycles of paternal X chromosome reactivation [14,15]. Our findings are consistent with this dynamic regulation of X-linked gene expression. Notably, PGCs migrating [16,17] from the embryonic clump showed similar GFP intensity to that observed in giant trophoblast cells, as also reported by Rebuzzini et al. [18]. forming the junctional zone, where reactivation of the paternal X chromosome has been reported by Hadjantonakis et al. [10].

Initially, it was proposed that P63 and P53 regulate overlapping gene sets; however, subsequent studies have demonstrated that these transcription factors exhibit both partially redundant and unique functions [19]. Beyond its role in germ cell specification, P63 is implicated in diverse cellular processes, including tumour suppression or promotion, depending on the cellular context [12,20].

Consistent with previous reports [7], we observed variable levels of GFP expression across tissues. Cells and tissues with higher haemoglobin content, such as liver and lung, displayed weaker GFP fluorescence, possibly due to quenching effects associated with haemoglobin absorption [21]. GFP may not be detectable in the mouse liver even when the transgene is present, primarily due to tissue-specific promoter activity and epigenetic silencing, as ubiquitous promoters such as CAG or CMV can be partially repressed in hepatocytes by DNA methylation and histone modifications [22]. In addition, the liver exhibits strong intrinsic autofluorescence caused by endogenous fluorophores, including flavins, NADH, and lipofuscin, which can mask weak GFP signals [23].

The high expression levels detected in the brain, heart, and germ cells within the gonads are driven by the CAG promoter present in the transgene construct, which contains multiple transcription factor binding sites and supports robust tissue-specific expression [24,25,26,27,28].

X-chromosome inactivation (XCI) is established during early development and is generally maintained throughout subsequent embryonic cell divisions. An exception to this stability occurs in primordial germ cells (PGCs), where epigenetic reprogramming leads to the reactivation of the previously silenced X chromosome. Although communication between PGCs and surrounding somatic cells is considered crucial for proper germ cell development, the underlying mechanisms of these interactions remain largely unclear [29].

Our fibroblast culture-based studies further indicate that the system may be suitable for studying tissue-specific promoter activity in in vitro differentiation models.

The differences in expression levels reflect the genomic localization of the transgene. In the EGFP strain, the reporter is autosomally integrated, resulting in uniform expression in nearly all cells. In contrast, in the XGFP strain, the transgene is located on the X chromosome and is subject to random XCI, in which either the maternal or paternal X chromosome is silenced with approximately equal probability. Consequently, only about half of the cells in female CD-1 × XGFP cultures are expected to express GFP. This mosaic pattern was confirmed by confocal microscopy, which demonstrated that only a subset of cells in female CD-1 × XGFP fibroblast cultures were GFP-positive. As expected, no GFP expression was detected in fibroblast cultures derived from male CD-1 × XGFP embryos, which inherit the Y chromosome from the father and therefore lack the X-linked GFP transgene.

Despite the usefulness of the CD-1 × XGFP model for monitoring paternal X-chromosome inactivation during early development, several limitations of the present study should be acknowledged [30]. First, GFP expression provides an indirect readout of X-chromosome activity and does not fully capture the molecular complexity of XCI regulation, including Xist RNA coating, histone modifications, and DNA methylation dynamics. Therefore, additional epigenetic analyses would be required to confirm the precise status of X-chromosome silencing or reactivation. Embryonic fibroblasts provide a convenient in vitro model; however, culture conditions may alter the epigenetic state of cells and may not completely reflect in vivo X-chromosome regulation. Future studies combining reporter-based monitoring with molecular approaches will be important for further validating and extending these findings.

## 5. Conclusions

In conclusion, our findings demonstrate that GFP expression varies among organs and tissues, providing valuable insights into tissue-specific gene activity, transplantation behaviour, and clonal dynamics. Confocal laser scanning microscopy is a suitable approach for mapping EGFP expression at single-cell resolution across diverse tissues.

## Figures and Tables

**Table 1:** Number of used E3.5 blastocyst stage embryos.

Number of Used Blastocyst Stage Embryos				
CD-1 × XGFP		CD-1 × EGFP	XGFP × XGFP	EGFP × EGFP
Female	Male			
15	14	15	22	30

**Table 2:** Number of used 14.5 dpc blastocyst stage embryos.

Number of Used 14.5-Day-Old Embryos				
CD-1 × XGFP		CD-1 × EGFP	XGFP × XGFP	EGFP × EGFP
Female	Male			
28	210	14	8	24

**Table 3:** Selected embryos to establish the observed MEF cultures.

Established Embryonic Fibroblast Cell Lines					
CD-1 × XGFP		CD-1 × EGFP		EGFP × EGFP	
Female	Male	Female	Male	Male	Female
CD1-XGFP-7	CD1-XGFP-9	CD1- EGFP-Ia	CD1- EGFP-IIa	EGFP-EGFP-1	EGFP-EGFP-2

**Table 4:** The following tables contain the information on the observed mouse embryonic fibroblast cell lines (MEF). Cell lines were tested with NanoEntek Arthur Fluorescents cell counter for average cell number, cell size, the ratio of GFP expressing cells, and average expression intensity.

**MEF Cell Lines**	**EGFP-EGFP-1♂ (A)**	**CD-1-EGFP-IIa♂ (B)**	**CD-1-XGFP-9♂ (C)**
Cell number (cell/µL)	6.32 × 10^5^	4.69 × 10^5^	2.06 × 10^6^
Green cell number (cell/µL)	5.78 × 10^5^	4.05 × 10^5^	0.05 × 10^4^
% green cells	91	86	0
average green intensity (RFU) ± SD	2725 ± 72	2829 ± 85	982 ± 6
average cell size (µm)	15	17	15
**MEF Cell Lines**	**EGFP-EGFP-2** **♀ (D)**	**CD-1-EGFP-Ia** **♀ (E)**	**CD-1-XGFP-7** **♀ (F)**
Cell number (cell/µL)	1.39 × 10^6^	2.02 × 10^6^	1.19 × 10^6^
Green cell number (cell/µL)	1.30 × 10^6^	1.93 × 10^6^	1.49 × 10^6^
% green cells	94	95	56
average green intensity (RFU) ± SD	2800 ± 36	2853 ± 36	1952 ± 38
average cell size (µm)	16	17	16

## Data Availability

The original contributions presented in this study are included in the article/Supplementary Material. Further inquiries can be directed to the corresponding author.

## Supplementary Materials

The following supporting information can be downloaded at https://www.mdpi.com/2218-273X/16/3/375/s1. Figure S1: Comparison of GFP expression in 14.5 dpc embryos; Figure S2: Confocal images of GFP expression in MEF cultures; Table S1: Primer sequences for sex determination in MEF cultures; Movie S1: Z-stack video of the GFP expression in CD-1 × EGFP embryos; Movie S2: Z-stack video of the GFP expression in CD-1 × XGFP embryos.

## Abbreviations

The following abbreviations are used in this manuscript:

DMSO	Dimethyl sulfoxide
dpc	Day post coitum
FBS	Foetal bovine serum
FM	Fibroblast medium
EGFP	Enhanced green fluorescent protein
GFP	Green fluorescent protein
gTB	Giant trophoblast cell
ICM	Inner cell mass
MEF	Mouse embryonic fibroblast
PFA	paraformaldehyde
PGC	Primordial germ cell
XCI	X-chromosome inactivation
XGFP	X-linked green fluorescent protein
