# Supplementary material for: X-Linked EGFP Reporter as a Tool to Examine X-Chromosome Inactivation in Mouse Embryos and Embryonic Fibroblasts
Source: Biomolecules. 2026 Mar 2;16(3):375. doi: 10.3390/biom16030375 (PMC13319934; doi:10.3390/biom16030375)
Supplement: Supplementary file 1 [file biomolecules-16-00375-s001.zip › biomolecules-4138455-supplementary.pdf]

biomolecules

Aritcile

X-Linked EGFP Reporter as a Tool to Examine X-Chromosome Inactivation in Mouse Embryos and Embryonic Fibroblasts

Martin Urbán [1,2,3], András Ecker [1,2,4], Roland Imre Tóth [1,2], Bence Lázár [1,2,5], Szilárd Bodó [6] and Elen Gócza [1,2,*]

1 Institute of Genetics and Biotechnology, Hungarian University of Agriculture and Life Sciences, Szent-Györgyi Albert str. 4, 2100 Gödöllő, Hungary; urban.martin@ttk.hu (M.U.); ecker.andras@uni-mate.hu (A.E.); gyongybagoly89@gmail.com (R.I.T.); lazar.bence@uni-mate.hu (B.L.)
2 Agribiotechnology and Precision Breeding for Food Security National Laboratory, 2100 Gödöllő, Hungary
3 Institute of Molecular Life Sciences, Center of Excellence of The Hungarian Academy of Sciences, HUN-REN Research Centre for Natural Sciences, 1117 Budapest, Hungary
4 Institute of Aquaculture and Environmental Safety, Hungarian University of Agriculture and Life Sciences, Szent-Györgyi Albert str. 4, 2100 Gödöllő, Hungary
5 National Centre for Biodiversity and Gene Conservation, 2100 Gödöllő, Hungary
6 Department of Animal Husbandry Sciences, Hungarian University of Agriculture and Life Sciences, 2100 Gödöllő, Hungary; bodo.szilard@uni-mate.hu
* Correspondence: gocza.elen@uni-mate.hu; Tel.: +36-30-300-4967

Table S1. Mouse embryonic fibroblast (MEF) cultures were established from embryos at 14.5 dpc.

Gene	Primer	Sequence (5'-3')	Product Size (bp)	GenBank Accession
Zfx	Zfx_L	AACATCCTGAACACCTTGCC	104	NM_011768
Zfx	Zfx_R	TAGCTTGTGGCTCTCCAGGT		NM_011768
Zfy	Zfy_L	CCATCAGCACTCAAAAAGCA	299	X14382
Zfy	Zfy_R	GCCTTTGTGTGAACGGAAAT		X14382

Table S1 shows the sequences of the primers used for sex determination of the fibroblast cultures.

 https://doi.org/10.3390/xxxxx

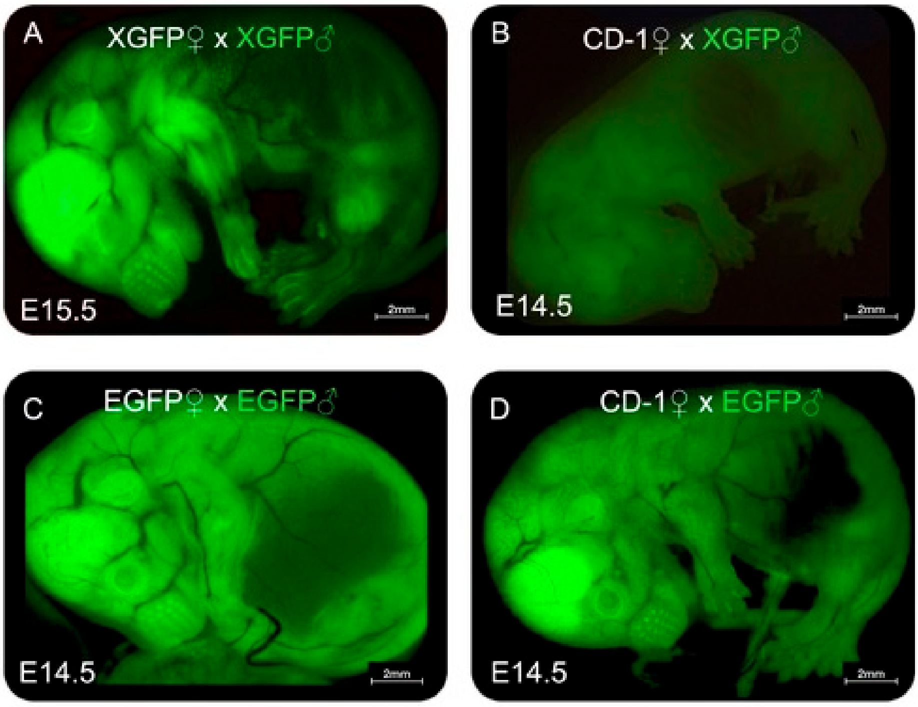

Figure S1. Comparison of GFP expression in 14.5 dpc embryos. In 14.5-day-old embryos, clear differences in the intensity of GFP expression were observed among genotypes. When XGFP × XGFP embryos were compared with EGFP × EGFP embryos, significantly stronger and more homogeneous GFP expression was observed in EGFP × EGFP embryos under identical microscope settings. Similarly, CD-1 × EGFP embryos exhibited markedly higher GFP fluorescence than CD-1 × XGFP embryos.

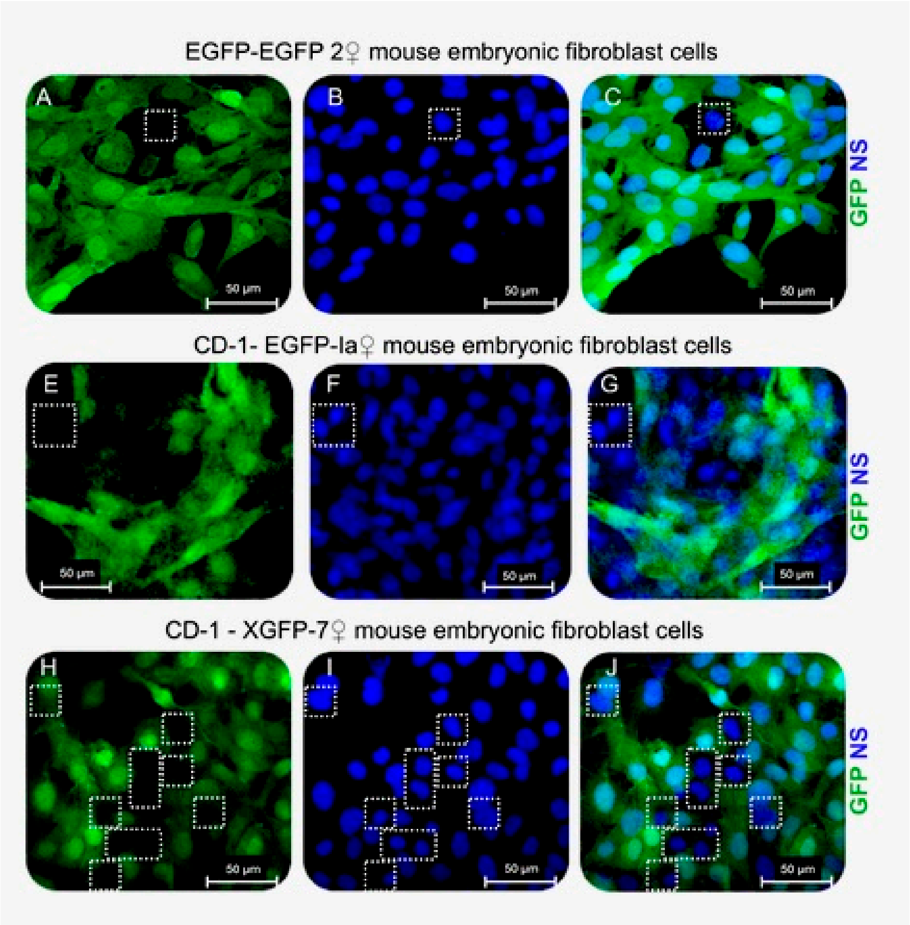

:

Figure S2. Confocal images of GFP expression in MEF cultures. Mouse embryonic fibroblast (MEF) cultures were established from embryos at 14.5 dpc and expanded by serial passaging. For immunostaining and GFP expression analysis, a subset of cells was seeded onto 0.1% gelatine-coated slides. Cells were fixed in 4% paraformaldehyde (PFA) and subsequently stained with TO-PRO-3 iodide as a nuclear counterstain. Samples were examined using a Leica TCS SP8 confocal microscope.

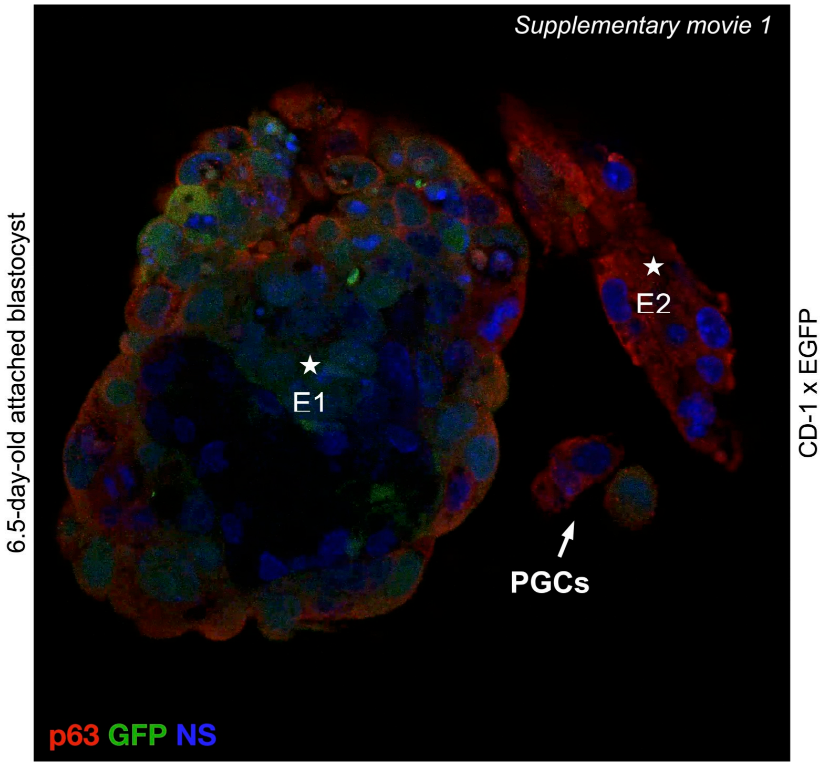

Movie S1. GFP expression in CD-1 x EGFP embryos**.** Following microscopic examination, embryos were transferred to culture dishes coated with 0.1% gelatine and further cultured in embryonic stem cell medium. At 6.5 dpc, mouse embryos attached to the MEF feeder layer. PGCs exhibited high levels of GFP expression and showed positive P63 immunostaining. In EGFP embryos, fluorescence intensity was reduced but remained clearly detectable (E1, E2). https://drive.google.com/file/d/1YZHCvhN9GNvnR3FCDq416xVt9t96mEZM/view?usp=share_link.

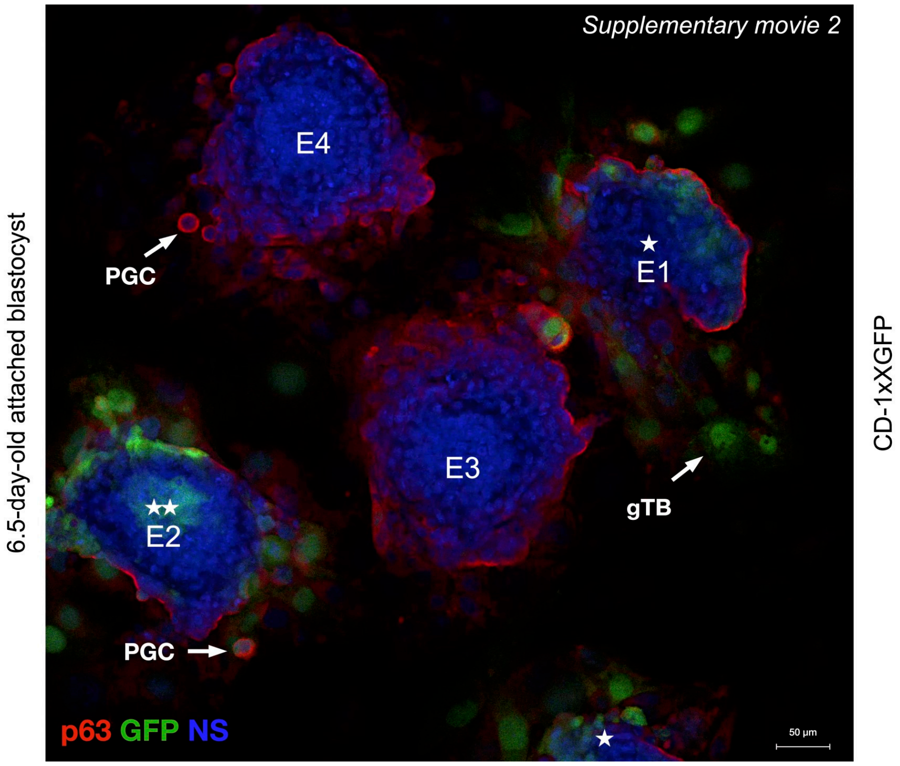

Movie S2. GFP expression in CD-1 x XGFP embryos. Following microscopic examination, embryos were transferred to culture dishes coated with 0.1% gelatine and further cultured in embryonic stem

cell medium. At 6.5 dpc, mouse embryos attached to the MEF feeder layer. In XGFP embryos (E1, E2, E3, E4), only minimal GFP expression was observed in extraembryonic tissues. GFP expression was detected in the female embryos (E1, E2) in both the trophoblast (gTB) and the epiblast (**). Large, round cells exhibited high levels of GFP expression; however, gTB cells did not show p63 positivity. A small population of round cells located at the periphery of the attached ICM showed strong co-expression of GFP and P63. Based on their morphology and P63 positivity, round cells migrating from the inner cell mass (ICM) are presumed to represent PGCs. https://drive.google.com/file/d/1TSmafD-Tb3gz6ycnBacnzgoMHg2tp8TP/view?usp=share_link
